# PhyloWGS: Reconstructing subclonal composition and evolution from whole-genome sequencing of tumors

**DOI:** 10.1186/s13059-015-0602-8

**Published:** 2015-02-13

**Authors:** Amit G Deshwar, Shankar Vembu, Christina K Yung, Gun Ho Jang, Lincoln Stein, Quaid Morris

**Affiliations:** Edward S Rogers Sr Department of Electrical and Computer Engineering, University of Toronto, Toronto, Canada; The Donnelly Center, University of Toronto, Toronto, Canada; Informatics & Biocomputing Program, Ontario Institute for Cancer Research, Toronto, Canada; Department of Computer Science, University of Toronto, Toronto, Canada; Department of Molecular Genetics, University of Toronto, Toronto, Canada

## Abstract

Tumors often contain multiple subpopulations of cancerous cells defined by distinct somatic mutations. We describe a new method, PhyloWGS, which can be applied to whole-genome sequencing data from one or more tumor samples to reconstruct complete genotypes of these subpopulations based on variant allele frequencies (VAFs) of point mutations and population frequencies of structural variations. We introduce a principled phylogenic correction for VAFs in loci affected by copy number alterations and we show that this correction greatly improves subclonal reconstruction compared to existing methods. PhyloWGS is free, open-source software, available at https://github.com/morrislab/phylowgs.

**Electronic supplementary material:**

The online version of this article (doi:10.1186/s13059-015-0602-8) contains supplementary material, which is available to authorized users.

## Background

Tumors contain multiple, genetically diverse subclonal populations of cells that have evolved from a single progenitor population through successive waves of expansion and selection [1-3]. Reconstructing their evolutionary histories can help identify characteristic driver mutations associated with cancer development and progression [4,5], and can provide insight into how tumors might respond to treatment [6,7]. In some cases, it is possible to genotype the subpopulations present in a tumor, while reconstructing its history, using the population frequencies of mutations that distinguish these subclonal populations [2,8-21]. Increasingly, tumors are being characterized using whole-genome sequencing (WGS) of bulk tumor samples [22] and few automated methods exist to perform this reconstruction on the basis of these data reliably.

Subclonal reconstruction algorithms attempt to infer the population structure of heterogeneous tumors based on the measured variant allelic frequency (VAF) of their somatic mutations. Some methods perform this reconstruction based solely on single nucleotide variants or small indels (collectively known as *simple somatic mutations* or SSMs) [16-19,21,23]. Others use changes in read coverage to identify genomic regions with an average ploidy that differs from normal, which they explain using inferred copy number variations (CNVs) that affect some of the cells in the sample [15,20,24,25].

The low read depth of current WGS complicates subclonal reconstruction. Until recently, subclonal populations (i.e., *subpopulations*) were defined based on accurate estimates of the proportion of cells with each mutation (i.e., their *population frequency*), which, for individual SSMs, are only available through targeted resequencing where the read depths are orders of magnitude higher than typical WGS depths [17,18,23]. However, preliminary evidence suggests that the much larger number of mutations detected by WGS can compensate for their decreased read depth [26]. In contrast, CNVs affect large, multi-kilobase-sized or megabase-sized regions of the genome, which allow the average copy number of these regions to be accurately estimated with WGS. Unfortunately, CNV-based subclonal reconstruction is more difficult than SSM-based reconstruction because of the need to estimate simultaneously population frequency and new copy number for each CNV. Most CNV-based methods only attempt to infer the copy number status of the *clonal* cancerous population [24,25] that contains the mutations shared by all of the cancerous cells. The few CNV-based methods [15,20] that attempt to resolve more than one cancerous subpopulation are practically limited to a small number (often two) of subpopulations. In contrast, SSM-based methods applied to targeted resequencing data can reliably resolve many more cancerous subpopulations [16-18,23]. However, it remains unclear what the limits of WGS-based automated subclonal reconstruction are.

Another open question is how to combine CNVs and SSMs when doing reconstruction. CNVs overlapping SSMs can interfere with SSM-based reconstruction because they complicate the relationship between VAF and population frequency. Although some methods attempt to model the impact of CNVs on the allele frequency of overlapping SSMs [17-19,27], these methods have significant restrictions. For example, several of these methods [17,18] make the unrealistic assumption that every cell either contains the structural variation and the mutation or neither. Also, no method places structural variations in a phylogenetic tree, which is important for studying the evolution of cancerous genomes.

We describe PhyloWGS, the first method designed for complete subclonal phylogenic reconstruction of both CNVs and SSMs from WGS of bulk tumor samples. Unlike all previous methods, PhyloWGS appropriately corrects SSM population frequencies in regions overlapping CNVs and is fast enough to perform reconstruction of at least five cancerous subpopulations based on thousands of mutations. We present results on subclonal reconstruction problems that cannot be correctly reconstructed using previous methods. We also probe the relationship between WGS read depth and the number of subpopulations that PhyloWGS can recover. Finally, we demonstrate that even in the absence of reliable CNV estimates, it is still feasible to perform automated subclonal composition reconstruction based on SSM frequency data at typical WGS read depths (30 to 50 ×), even for highly rearranged genomes where less than 2*%* of the SSMs lie in regions of normal copy number. Open-source, free software implementing PhyloWGS is available under the GNU General Public License v3 [28].

### Previous work

**Figure 1:**
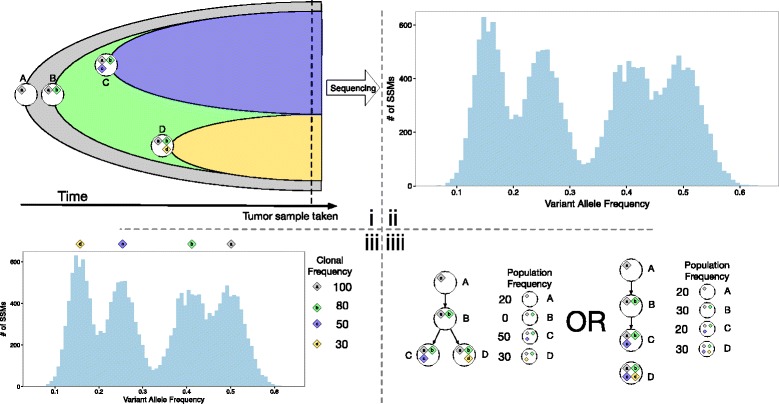
**The development of intratumor heterogeneity and subclonal reconstruction.** Tumor composition over time **(i)**, the resulting distribution of variant allele frequencies (VAFs) **(ii)**, the result of successful inference of the VAF clusters **(iii)**, and the desired output of subclonal inference **(iiii)**. SSM, simple somatic mutation; VAF, variant allelic frequency.

Figure 1 provides an overview of an evolving tumor, the measurement of somatic VAFs and the resulting subclonal reconstruction process. Panel (i) of this figure shows a visualization of the evolution of a tumor over time as non-cancerous cells (subpopulation A, shown in grey) are replaced by, at first, one clonal cancerous population (subpopulation B, shown in green), which then further develops into multiple cancerous subpopulations (C and D, shown in blue and yellow, respectively). Tumor cells define new subpopulations by acquiring new oncogenic mutations that allow their descendants to expand relative to the other tumor subpopulations. Each circle in panel (i) refers to a subpopulation. We associate subpopulations with the set of shared somatic mutations that distinguish it from its parent subpopulation (or, in the case of A, from the germ line (or reference) genome); this mutation set is indicated by the corresponding lower case letter (e.g. mutation set *b* first appears in subpopulation *B*). However, each subpopulation also inherits all of its parent’s mutations; the *subclonal lineage* of a mutation is the set of all subpopulations that contain it (e.g., the subclonal lineage of a is A, B, C and D).

In general, the subpopulation-defining mutation sets include more than one mutation. Cancerous cells often have increased mutation rates, and even non-cancerous cells accumulate somatic mutations at a rate of 1.1 per cell division [29]. As such, subpopulations are defined not only by the small number of oncogenic ‘driver’ mutations that support rapid expansion but also by a larger number of ‘passenger’ mutations acquired before the driver mutation(s). The selective sweeps that cause subpopulation expansion increase the population frequency of both driver and passenger SSMs, driving them to having indistinguishable population frequencies [30,31]. However, sampling and technical noise in sequencing means that the observed VAFs are distributed around the true value for a subpopulation. Panel (ii) shows an example histogram of measured VAFs for SSMs found in a heterogeneous tumor sample.

**Figure 2:**
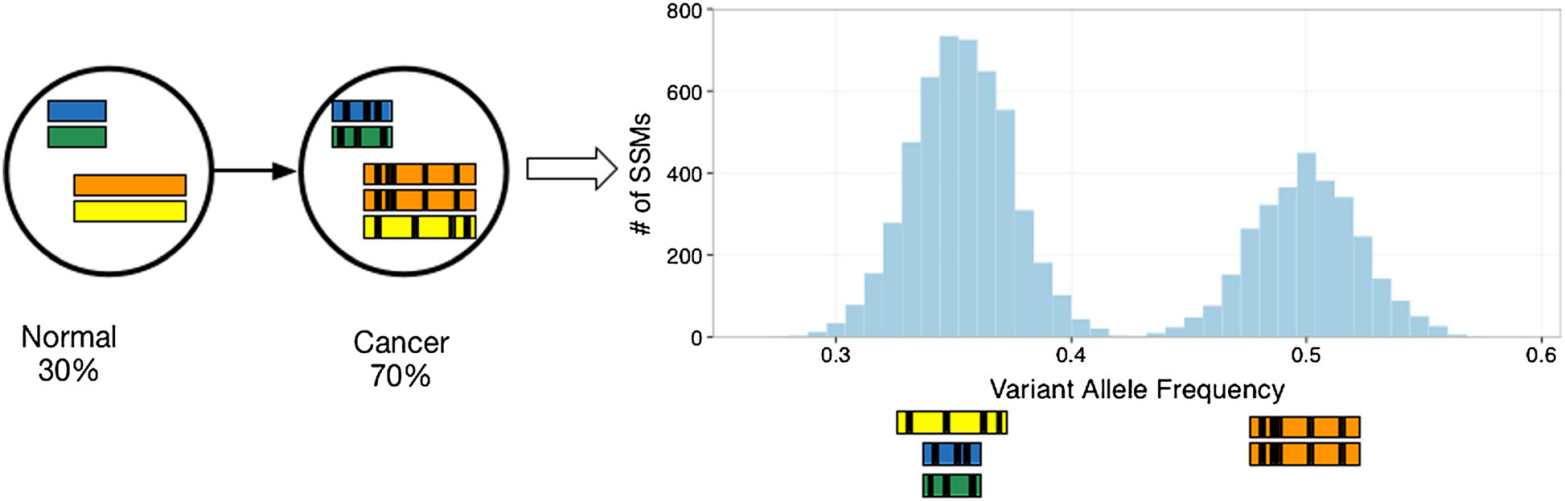
**Example of copy number variations affecting the distribution of variant allele frequencies.**

Subclonal reconstruction algorithms define mutation sets, and their associated subpopulations, by analyzing the population frequencies of somatic mutations detected in a tumor sample. In Figure 1, all mutations are SSMs, and all SSMs occur on one copy in diploid regions of the genome. In this case, the estimated population frequency of an SSM is simply twice its VAF. Figure 2, discussed in the next section, shows how CNVs overlapping SSM loci change this relationship. Note that although each VAF cluster corresponds to a subclonal lineage, and a subpopulation that was present at some point during the tumor’s evolution, this subpopulation need not be present when the tumor is sampled. In Figure 1, subpopulation B is no longer present in the tumor, although its two descendant subpopulations are. These *vestigial* VAF clusters, if they exist, always correspond to subpopulations at branchpoints in the phylogeny, however, not every branchpoint generates a vestigial cluster.

#### Simple-somatic-mutation-based approaches

SSM-based subclonal reconstruction algorithms attempt to reconstruct the subpopulation genotypes based on VAF clusters (and their associated mutation sets) identified by fitting statistical mixture models to the VAF data either without phylogenic reconstruction [18,19,21,32], before phylogenic reconstruction [33] or concurrently with it [16,17]. Often, as in Figure 1, the clusters overlap, which introduces uncertainty in the exact number of mutation sets represented in the tumor (as well as in the assignment of SSMs to clusters). Adding more clusters to the model always provides a better data fit, so to prevent overfitting, the cluster number is selected by balancing data fit versus a complexity penalty (e.g. the Bayesian information criteria) or by Bayesian inference in a non-parametric model [17,18,32]. In panel (iii) in Figure 1, the correct number of clusters has been recovered along with appropriate central VAFs.

Assuming that the correct VAF clusters can be recovered, the subclonal lineages corresponding to each mutation set must still be defined. Defining the subclonal lineages is equivalent to defining the tumor phylogeny, and often multiple phylogenies are consistent with the recovered VAF clusters (e.g. panel (iiii) in Figure 1). Complete and correct reconstruction of subpopulation genotypes requires resolving this ambiguity. To do so, reconstruction methods make one of a handful of assumptions about the process of tumor evolution.

A common, and powerful, assumption is the infinite sites assumption (ISA) [17,34,35], which posits that each SSM occurs only once in the evolutionary history of the tumor. The ISA implies that the tumor evolution is consistent with a ‘perfect and persistent phylogeny’ [18]: each subpopulation has all of the SSMs that its ancestors had, each SSM appears in only one subclonal lineage and each subclonal lineage corresponds to a subtree in the phylogeny of tumor subpopulations. Because SSMs are relatively rare (compared to the genome size), the ISA is nearly always valid for all SSMs, so there is little danger of incorrect reconstructions due to violations of the ISA. In many cases, the ISA alone permits the recovery of multiple, complete subpopulation genotypes from a single or small number of tumor samples using either the *sum rule* [17] (also called the pigeonhole principle [26]) or the *crossing rule* [17], respectively. Methods that do not use the ISA require, in the case of no measurement noise, at least as many tumor samples as there are subpopulations [16,36]; in actual application when there is noise, even more samples are required.

Unfortunately, the ISA alone is often unable to resolve reconstruction ambiguity fully. As such, some methods [16,33] also make a sparsity assumption to select among ISA-respecting phylogenies consistent with the VAF data. This assumption, which we call *strong parsimony*, posits that due to expansion dynamics, there are a small number of subpopulations still present in the tumor [16,33], and that many of the VAF clusters are vestigial. These methods therefore select the phylogeny (or phylogenies) that maximizes the number of vestigial VAF clusters [16], or equivalently, the number of branchpoints where the parental subpopulation has a zero frequency in the current tumor [16,33]. The strong parsimony assumption does resolve some ambiguity, and leads to the correct reconstruction in Figure 1, but it is risky as its empirical validity is not yet established. For example, under some conditions, a linear (i.e. non-branching) phylogeny can be mistaken for a branching one; the risk of this occurring increases as the VAF measurement noise or the number of subpopulations in the tumor increases. This background distribution of false positive vestigiality is not yet considered by either of the methods that assume strong parsimony.

By assigning all SSMs within a VAF cluster to the same mutation set, reconstruction methods make another implicit assumption, which we call *weak parsimony*. This assumption does not hold if two mutation sets have the same population frequency. Note that if the ISA is valid, by the pigeonhole principle, weak parsimony is guaranteed to be valid whenever the population frequency of the mutation set is >50%.

Table 1 classifies reconstruction methods based on these assumptions, whether they recover complete subpopulation genotypes (or simply identify subclonal lineages), and whether they can handle single tumor samples, multiple tumor samples or both.

**Table 1:** **Subclonal reconstruction methods, their properties and assumptions**

**Property/method**	**PhyloWGS**	**PhyloSub [** 17 **]**	**THetA [** 15 **]**	**PyClone [** 18 **]**	**TrAp [** 16 **]**	**Clomial [** 36 **]**	**RecBTP [** 33 **]**
Simple somatic mutation	Y	Y	N	Y	Y	Y	Y
Copy number variation	Y	N	Y	N	N	N	N
Weak parsimony	Y	Y	N/A	Y	Y	Y	Y
Strong parsimony	N	N	N/A	N	Y	N	Y
Infinite sites	Y	Y	N/A	Y	Y	N	Y
Phylogenetic inference	Y	Y	N	N	Y	N	Y
Parametric	N	N	Y	N	N	Y	N
Multiple samples	Y	Y	N	Y	N	Y	N
Genotype uncertainty	Y	Y	N	N/A	N	N	N

N/A, not applicable.

PhyloWGS, like its predecessor PhyloSub [17], does not make the strong parsimony assumption nor does it report only a single tree. Instead it reports samples from the posterior distribution over phylogenies. Because the clustering of the VAF is performed concurrently with phylogenic reconstruction, PhyloWGS is able to perform accurate reconstruction even when the weak parsimony assumption is violated in a strict subset of the samples available, for example, if the VAF clusters overlap in one sample but not another. Our Markov chain Monte Carlo (MCMC) procedure samples phylogenies from the model posterior that are consistent with the mutation frequencies and does not rule out phylogenies that are equally consistent with the data. From this collection of samples, areas of certainty and uncertainty in the reconstruction can be determined.

#### Copy-number-variation-based approaches

There are three major differences between CNV-based reconstruction and SSM-based reconstruction. First, because large regions of the genome are affected by CNVs and reads, mapping across the regions can be used to estimate average ploidy and accurate quantification of changes in average copy number can be achieved with much smaller read depths (as low as 5 to 7 ×) [15,37]. However, the other two differences make CNV-based subclonal reconstruction more difficult and less generally applicable compared with SSM-based methods. One difference is that the ISA is often invalid because CNVs affect large regions of the genome. As such, it is more common to see overlapping mutations in independent cells; these make the reconstruction problem more challenging. Even when only one CNV affects a given region, inferring its population frequency is still challenging because at least two values, population frequency *ϕ*∈(0,1) and non-negative integer copy number *C*, have to be simultaneously inferred from a single observed, non-normal average copy number *x*≠2. In particular, this equation,

$$x = \phi C + (1-\phi) 2, $$

always has at least two different solutions for *x*>1.

In the absence of other information, like B-allele frequencies [26], parsimony assumptions are relied upon to resolve reconstruction ambiguities. One strategy only attempts to reconstruct the cancerous, subclonal lineage [24,25] with the highest population frequency (also known as the clonal population). From this reconstruction, the proportion of cells in the tumor sample that are cancerous (i.e. the cellularity), as well as the CNVs that are shared by all cancerous cells in the tumor, can be inferred. However, this approach can fail when there are multiple subclonal populations, especially if they share few CNVs [15,20]. Methods that attempt to detect >1 cancerous subpopulation do so by balancing data fit with a complexity term that penalizes additional subpopulations [15,20]. So far, these methods seem to be practically limited to a small number of cancerous subpopulations (i.e., two), and cannot be applied to tumors with substantial rearrangements.

#### Combining simple somatic mutations and copy number variations

In loci affected by CNVs, computing the population frequency of an SSM from its VAF requires knowing whether the SSM occurred before, after or independently of the CNV. If the SSM occurred before the CNV, and CNV affects the copy number of the SSM, then computing its VAF also requires knowing the new number of maternal and paternal copies of the locus. Figure 2 illustrates a situation where incorporating CNV information is critical for subclonal reconstruction. Without CNV information, the two VAF peaks would be interpreted as two separate subclonal lineages. With CNVs, it becomes clear that the second peak is caused by the amplification of part of the genome that increases the VAF of all SSMs found in the region.

Some subclonal reconstruction methods simply ignore the impact that CNVs have on the relationship between SSM population and allele frequency [16,21]. Other methods that do account for the effect of copy number changes on SSM frequencies [17-19], do so by integrating over all the possible relationships between allele frequency and population frequency without using that the ISA, which was necessary to associate SSMs uniquely to subclonal lineages in the first place, constrains this relationship [26].

For the first time, we describe an automated method, PhyloWGS, which performs subclonal reconstruction using both CNVs and SSMs. By combining information from both CNVs and SSMs, and properly accounting for their interaction, we provide a more comprehensive and accurate description of a subclonal genotype.

## Results

In the following, we first provide a brief explanation of how PhyloWGS incorporates both SSMs and CNVs in phylogenic reconstruction by converting CNVs into pseudo-SSMs and performing subclonal reconstruction on the SSMs and pseudo-SSMs; full details are provided in the Materials and methods section. Then, we show an illustrative example where accounting for the effect of CNVs on SSMs permits the correct subclonal reconstruction of a tumor population whereas using either CNV or SSM data in isolation does not. Then, we describe our efforts to quantify the relationship between read depth and the number of subpopulations that can be accurately recovered by applying PhyloWGS to simulated WGS data with different read depths, number of subpopulations and SSMs. Next, we describe the application of PhyloWGS to three TCGA benchmark datasets. Finally, we describe the application of PhyloWGS to two real datasets: a multiple-sample WGS dataset from a patient with chronic lymphocytic leukemia and a single sample from a breast tumor.

### Incorporating copy number variations with simple somatic mutations in phylogenic reconstruction

We assume that a CNV algorithm has already been applied to the sequencing data and that this algorithm provides estimates of copy number *C* and population frequency *ϕ*_*i*_ for each CNV *i*. We use these estimates in two ways: first, for each CNV, we create an equivalent *pseudo-SSM* with population frequency *ϕ*_*i*_ by adding an SSM to the dataset with total reads *d*_*i*_ and variant reads *d*_*i*_×*ϕ*_*i*_/2 rounded to the nearest whole number (i.e., the expected number of variant reads of a heterozygous mutation with population frequency *ϕ*_*i*_) where *d*_*i*_ is set to a user-defined multiple of the average WGS read depth. If a confidence interval for *ϕ*_*i*_ is available, we can set *d*_*i*_ to have the same confidence interval. Note, we allow multiple CNVs to affect the same locus; each of these CNVs is assigned its own pseudo-SSM. Second, we associate all SSMs within the region affected by the CNV to this pseudo-SSM. Our model (described in the Materials and methods section) uses this association to compute the transformation from the inferred population frequency of an SSM to its expected VAF.

Here, we briefly describe this transformation when there is only one CNV affecting the SSM locus; the Materials and methods section describes the general version of the transformation used by PhyloWGS that allows multiple CNVs to affect the locus.

Given the population frequency of the CNV, *ϕ*_*c*_, the copy number of the CNV *C* broken down into maternal and paternal components *C*=*C*^*p*^+*C*^*m*^, and the population frequency of the SSM, *ϕ*_*s*_, the equations below compute the expected allele frequency of the SSM *x*_*ssm*_. Here we are using the terms ‘maternal’ and ‘paternal’ simply to distinguish the two copies and not to suggest that we have actually assigned each chromosome to each parent. Furthermore, the description here assumes that the SSM is on the maternal copy; if it is on the paternal copy, replace *C*^*m*^ with *C*^*p*^ below.

If an SSM lies in a region affected by a CNV, there are three possibilities for their phylogenic relationship:

1. The SSM precedes the CNV event, i.e., the CNV occurred in a cell already containing the SSM.
2. The SSM occurs after the CNV event, i.e., the SSM occurred in a cell already containing the CNV.
3. The SSM and CNV occurred in separate branches of the phylogeny, i.e., the mutations occur in separate cells and no cell contains both the SSM and the CNV.

#### Case 1: simple somatic mutation → copy number variation

Because of the ISAs, this phylogenic relationship requires *ϕ*_*s*_≥*ϕ*_*c*_>0. In this case, cells with the CNV contain *C*^*m*^ copies of the SSM, and cells with the SSM but not the CNV have only one mutated copy. As such, the expected allele frequency can be written as:

$$\begin{aligned} x_{ssm} = \frac{C^{m} \phi_{c} + (\phi_{s}-\phi_{c})}{2(1-\phi_{c}) + C\phi_{c}}. \end{aligned} $$

The numerator corresponds to the average number of copies per cell of the SSM-mutated locus in the population and the denominator is the average number of copies per cell of the locus (mutated or not) in the population. We note that if there is no copy number change in *C*^*m*^ then the numerator is simply *ϕ*_*s*_, and if *C*^*m*^=0 then the numerator is *ϕ*_*s*_−*ϕ*_*c*_.

#### Case 2: copy number variation → simple somatic mutation

This case is only possible if the maternal locus still exists after the CNV (i.e. *C*^*m*^≥1), and furthermore that *ϕ*_*c*_≥*ϕ*_*s*_>0. By the ISA, only one copy of the locus is affected, so the numerator is simply *ϕ*_*s*_ and we do not need to know the breakdown of *C* into *C*^*m*^ and *C*^*p*^. As such:

$$\begin{aligned} x_{ssm} = \frac{\phi_{s}}{2(1-\phi_{c}) + C\phi_{c}}. \end{aligned} $$

#### Case 3: $\genfrac {}{}{0pt}{}{\nearrow }{\searrow }^{SSM}_{\textit {CNV}}$

In this case, the SSM and CNVs lie on different branches of the phylogeny and no cell in the population contains both mutations, so the only constraints on *ϕ*_*s*_ and *ϕ*_*c*_ are that *ϕ*_*s*_+*ϕ*_*c*_≤1. As per Case 2, the average number of loci affected by the SSM is *ϕ*_*s*_. So the expected allele frequency is identical to Case 2:

$$\begin{aligned} x_{ssm} = \frac{\phi_{s}}{2(1-\phi_{c}) + C\phi_{c}}. \end{aligned} $$

**Figure 3:**
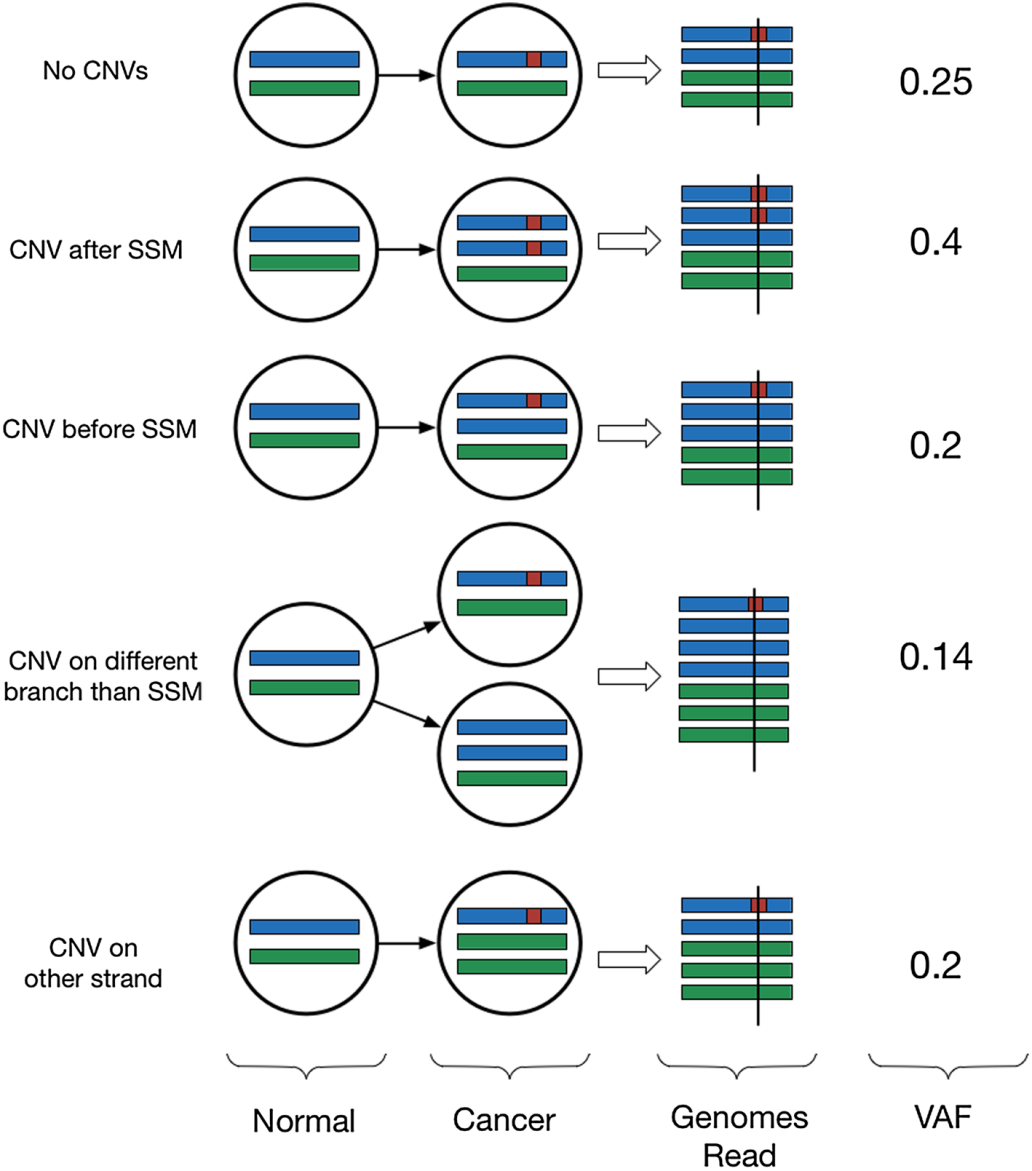
**Changes to VAF caused by CNVs with different phylogenetic relationships.** CNV, copy number variation; SSM, simple somatic mutation; VAF, variant allelic frequency.

We illustrate some of the ways in which the relationship between a CNV and an affected SSM in the phylogenetic tree affects the observed VAF of that SSM in Figure 3.

We note that the breakdown of *C* into *C*^*m*^ and *C*^*p*^ and phasing the SSM only affects the expected VAF in Case 1. This is because it is the only case where a CNV event can affect a mutated locus. Although PhyloWGS requires the breakdown of *C* into *C*^*m*^ and *C*^*p*^ under these conditions, we do not require the SSM to be phased, as many cannot be [26], and instead consider both possibilities when computing the likelihood. Some subclonal copy number callers decompose *C* into *C*^*m*^ and *C*^*p*^ [38]; if the caller does not provide this decomposition, then PhyloWGS should be run on loci where *C*∈{0,1,2}.

An important consequence of these rules is that under some conditions, it is possible to identify unambiguously a branching phylogeny using single sample data. If an SSM can be phased to an amplified locus there are situations where given particular values of *x*_*ssm*_, *ϕ*_*c*_, *C*^*p*^ and *C*^*m*^ one can distinguish between Case 1 and Case 3. For example, given *x*_*ssm*_=0.1,*ϕ*_*c*_=0.4,*C*^*m*^=10 and *C*^*p*^=1, for Case 3 the inferred *ϕ*_*s*_ is 0.56. However, if Case 1 were true, the resulting inferred *ϕ*_*s*_ would be negative and so Case 1 is not possible. This condition holds whenever *x*_*ssm*_×(2(1−*ϕ*_*c*_)+*Cϕ*_*c*_)<(*C*^*m*^−1)*ϕ*_*c*_. We were unable to find any other circumstances in which single sample VAFs were more consistent with a branching phylogeny than a chain phylogeny.

### Combination of copy number variations with simple somatic mutations is required for accurate subclonal reconstruction

**Figure 4:**
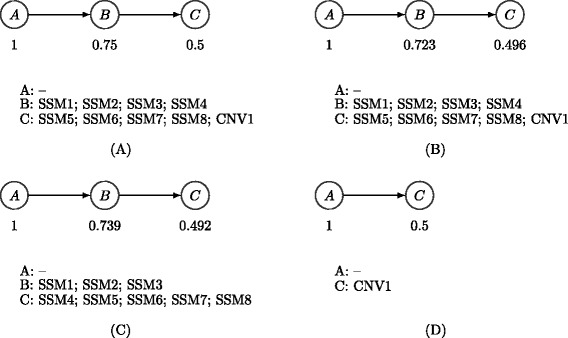
**Example subclonal structure and inferred phylogenies using different methods.** **(A)** Example of tumor subclonal structure. **(B)** Tumor phylogeny recovered by PhyloWGS. **(C)** Tumor phylogeny recovered by PhyloSub. **(D)** Subclonal structure implied by only CNVs. CNV, copy number variation; SSM, simple somatic mutation.

Consider a tumor where 25% of the cells are normal (no SSMs and diploid, population A), 25% come from a subpopulation with only SSMs (SSM1 to 4, population B) and 50% belong to a descendant subpopulation of B containing all the SSMs from B and adding new simple somatic variants (SSM5 to 8) and a homozygous deletion (CNV1) in the region containing SSM4, labeled population C. The evolutionary tree of this population is shown in Figure 4A. In reads sampled from this population, the expected VAFs for SSM1 to 3 are 37.5% (i.e. half of their population frequency) and for SSM5 to 8 they are 25%; however, based on the rules described in the Materials and methods section, the expected VAF of SSM4 is 25%. This is because all the copies of the genome at that position come from population A or B. Populations A and B are present in equal proportions and only one copy in population B contains variant reads, so 25% of the genomes contain the variant allele. As such, methods that do not incorporate the CNV change at the SSM4 locus will incorrectly assign SSM4 to population C. Also, methods that incorporate only CNV information cannot detect the subpopulation B, which is defined by SSM alone.

We generated simulated variant and reference allele counts for this example at a simulated read depth of 60. The reference and total read counts for each SSM can be found in Table 2. PhyloWGS was able to reconstruct correctly the evolutionary history and subpopulation structure (Figure 4B). However, a version of PhyloSub that ignored CNVs incorrectly assigned SSM4 to population C (Figure 4C). Furthermore, by construction, there is no way to recover population B based only on CNV data, so a perfect CNV-based algorithm would infer the subclonal structure in Figure 4D.

**Table 2:** **Reference and total read counts for example tumor sequencing data**

**Mutation id**	**Reference counts**	**Total counts**
s0	23	47
s1	35	69
s2	26	51
s3	29	56
s4	30	40
s5	42	70
s6	27	41
s7	36	56
s8	59	75
s9	57	76
s10	51	68
s11	37	51

We also ran PyClone [18] on this dataset. PyClone cannot take as input that a locus has been homozygously deleted, so we ran PyClone either by telling it there were no CNV changes or that there was a deletion of one copy. Without any input CNV alterations, PyClone produced a clustering identical to PhyloSub, while in the single copy deletion state, PyClone placed SSM4 in an additional cluster with no other mutations. As this simple example illustrates, integrating data from both SSMs and CNVs is required for full, and accurate, subclonal reconstruction.

### Applying PhyloWGS to simulated data

**Figure 5:**
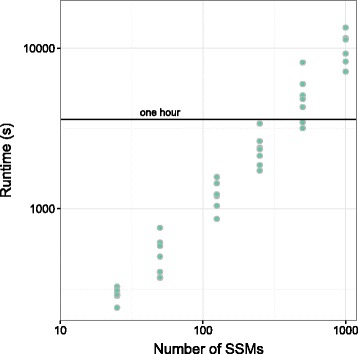
**PhyloWGS run time.** Relationship between the number of SSMs in the simulated dataset with five subpopulations and the run time on a log10 vs log10 plot. Run time was measured using a single core of an Intel i7-4770K with 2,500 MCMC iterations and 5,000 inner Metropolis–Hastings iterations. The run time can be greatly decreased by parallelizing the sampling or by taking less samples; however, the implications of these options have not been explored. SSM, simple somatic mutation.

An important question in subclonal analysis of tumor samples is estimating how deep sequencing must be to recover the subclonal structure. To answer this question, we applied PhyloWGS to simulated read counts with known subclonal structure. Our simulations looked at a range of total population counts (3, 4, 5 and 6), read depths (20, 30, 50, 70, 100, 200 and 300) and number of SSMs per population (5, 10, 25, 50, 100, 200, 500 and 1,000). For each combination of population count, read depth and SSMs per population, we generated simulated tumor data for which the subclonal population frequencies were consistent with both branching and linear phylogenies. For each simulated SSM *k* in subpopulation *u*, reference allele reads (*a*_*k*_) were drawn as:

$$ \begin{aligned} a_{k} \sim \text{Binomial}(d_{k}, 1-\phi_{u} + 0.5 \phi_{u}); \quad d_{k} \sim \text{Poisson}(r), \end{aligned} $$

where *ϕ*_*u*_ is the clonal frequency of population *u* and *r* is the simulated read depth. The *ϕ* values used for the simulations can be found in Table 3. First, we examined the time needed to complete sampling as a function of the number of SSMs (shown in Figure 5). In less than 3 hours on a single core of an Intel i7-4770K, on average, the inference could be completed with up to 1,000 SSMs (all timing data shown use the simulated dataset with five subpopulations).

**Table 3:** **Subclonal lineage proportions used**

**Number of populations**	***ϕ*** **values used (linear)**
3	0.44, 0.11
4	0.56, 0.25, 0.06
5	0.64, 0.36, 0.16, 0.04
6	0.71, 0.44, 0.25, 0.11, 0.03

**Figure 6:**
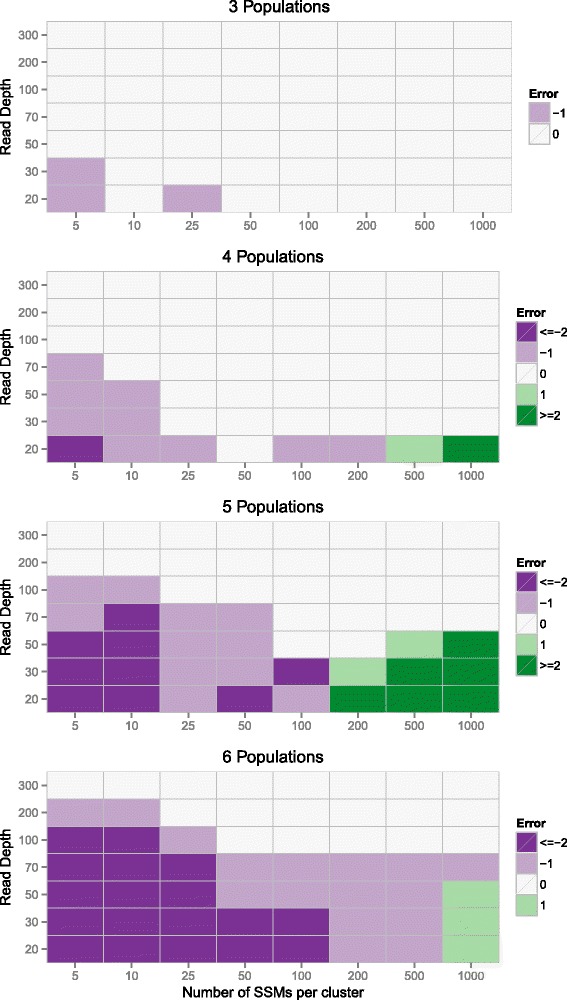
**Recovering the true number of clusters.** Each panel shows the relationship between the number of SSMs per cluster, the read depth and the ability of PhyloWGS to recover the true number of populations for simulations with three, four, five or six populations. The error is calculated by subtracting the true number of subclonal lineages from the number found. SSM, simple somatic mutation.

To determine the number of subpopulations our algorithm found, we analyzed the sampled tree with the highest complete data likelihood and removed any subpopulations with zero assigned SSMs. We then compared the difference between the number of subpopulations used to generate the data and the number of subpopulations identified by our algorithm. The results of this comparison for ambiguous phylogeny simulations are shown in Figure 6. Several relationships between simulation parameters and the output of our model can be observed. First, unsurprisingly, increasing the read depth and decreasing the number of subpopulations resulted in increased accuracy in the estimated number of subpopulations. Second, for the ambiguous phylogeny simulations, there is a U-shaped relationship between accuracy and the number of SSMs characterizing each population, where accuracy first increases and then decreases as the number of SSMs increases. This decrease in accuracy with high numbers of SSMs is unintuitive, since more SSMs provide more information with which to perform inference. However, the Dirichlet process prior sometimes overestimates the number of source components [39]. While this overestimation has not been demonstrated for the tree-structured stick-breaking process prior used by PhyloWGS, the similarity between the processes makes it likely that this is the case. While some of these errors can be eliminated by ad hoc removal of clusters with a small number of SSMs, there is not yet a consistent approach to do this, so we leave the results untouched. These results suggest that for three or four subpopulations, a read depth consistent with typical WGS experiments (20 to 30 ×) is sufficient to identify the correct number of subpopulations, while experiments with 200 to 300 × are needed to resolve tumors with up to six subpopulations.

Another important measure of the performance of our algorithm is how accurate the mapping from population to SSM is. To evaluate this accuracy in a systematic way that accounts for class imbalance, varying number of SSMs and differing number of clusters, we examine the area under the precision–recall curve (AUPRC) between the known true co-clustering matrix and the average co-clustering matrix from our samples. A co-clustering matrix *M* is a binary matrix where *M*_*ij*_=1 if SSM *i* and SSM *j* are in the same cluster. The average co-clustering matrix is constructed by taking the average of the co-clustering matrices of each sample in the Markov chain after burn-in and is an estimate of the posterior mean co-clustering matrix of our model. The average co-clustering matrix better predicts the true co-clustering matrix than the co-clustering matrix computed from the maximum data likelihood tree. AUPRC was chosen over area under the receiver–operator curve as it is known to be more informative in the presence of class imbalance [40], which changes as the number of populations increases.

**Figure 7:**
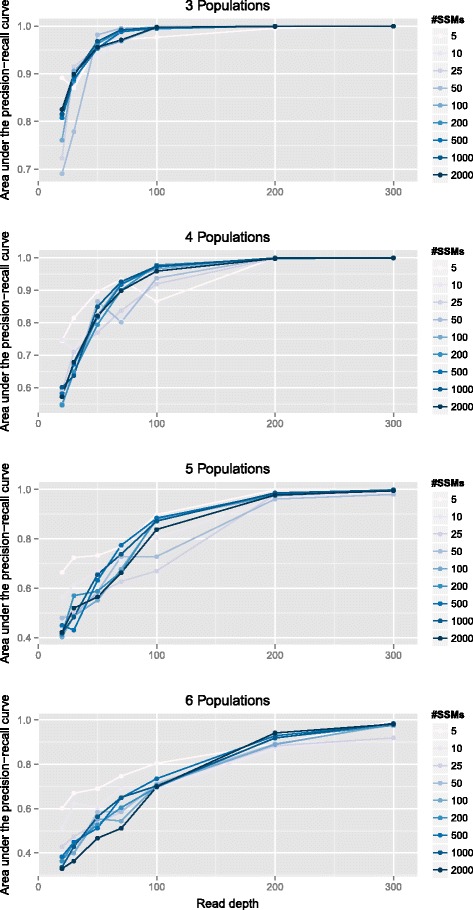
**Reconstruction accuracy.** Each panel shows the relationship between the read depth and the accuracy of the resulting clustering, measured as the area under the precision–recall curve (AUPRC). Plots for three, four, five and six populations are shown with each line representing a different number of SSMs per cancerous population. SSM, simple somatic mutation.

In Figure 7, we plot the resulting AUPRC for our simulation experiments. As with inferring the number of populations, our method does better as the read depth increases and the number of populations decreases. Unlike the last result, there is no clear relationship between the number of SSMs and the resulting AUPRC. To provide qualitative guidance to users of the meaning of various AUPRC cutoffs, we show several examples of inferred co-clustering matrices with AUPRCs of 0.65, 0.8, 0.9 and 0.98 in Additional file 1.

### Simulations with copy number variation changes

**Figure 8:**
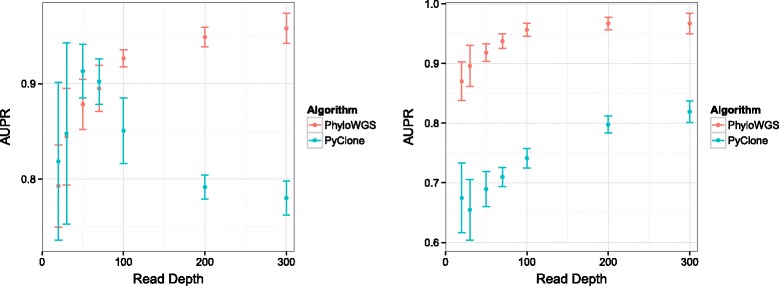
**Relationship between read depth and accuracy of the resulting clustering.** These were measured as the area under the precision–recall curve for PhyloWGS and PyClone. Plots are shown for subclonal additions (left) and deletions (right). AUPR, area under the precision–recall curve.

Next, we generated simulated data for a more complex genetic environment. In these cases we simulated data from a tumor with 20% normal tissue, a 40% CNV-free subpopulation with 500 mutations and a descendant subpopulation with another 200 mutations but a substantial CNV affecting 50% of the genome, either an amplification or a deletion. We simulated data with read depths of 20, 30, 50, 70, 100, 200 and 300, ten times for each read depth and alteration pair. We then applied PhyloWGS and computed the AUPRC scores. To demonstrate the importance of incorporating CNVs in phylogenetic reconstruction, we compared the scores from PhyloWGS with those from PyClone [18]. The performance of both methods can be seen in Figure 8. Using PhyloWGS results in superior clustering compared to PyClone for both subclonal amplifications and deletions, with the exception of amplifications with low read depths, where the performance distributions closely overlap.

### TCGA benchmark

**Figure 9:**
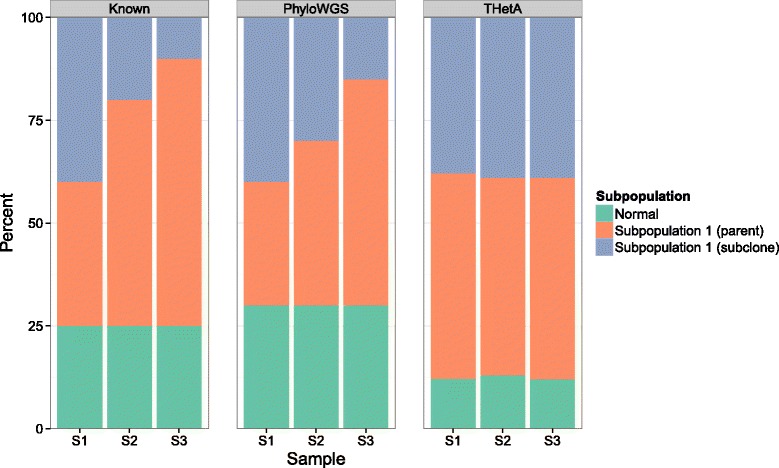
**True and inferred composition of TCGA benchmark samples.** The figure shows the true (left), inferred by PhyloSub (center) and inferred by THetA (right) composition of three TCGA benchmark samples. Each bar represents a single sample.

Next, we applied PhyloWGS to the TCGA variant-calling benchmark 4 dataset [41]. The samples we examined consist of a normal population, a cancerous cell-line population HCC 1143 and a spiked-in subclonal descendant of the cancerous population in various proportions with 30 × coverage. Starting with the publicly available BAM files, we identified locations of possible structural variation using BIC-seq [37] with default parameters, except for the bandwidth parameter, which was set to 1,000. We changed the bandwidth parameter because we found the default value of 100 resulted in overly noisy segmentations and highly variable normalized read counts. To identify SSMs and the number of variant and reference reads for each SSM, we reverted the BAM files into unaligned reads using Picard 1.90 [42]. Reads for each sample were then realigned using BWA 0.6.2 [43] and collapsed using Picard. Aligned reads of a cancerous sample and its matched normal were analyzed by two somatic calling tools: MuTect 1.1.4 [44] and Strelka 1.0.7 [45]. A set of high confidence mutations were extracted by taking an intersection of the calls made by MuTect and Strelka. Previous verification with other tumor/normal pairs showed that this approach achieved >90% precision (data not shown). We first ran THetA [15] using the output of BIC-seq with the aim of using THetA’s output to provide us with the CNV information that PhyloWGS requires (see Materials and methods section). However, despite that the subclonal population varied from 40% to 10%, THetA returned nearly identical composition inferences for all the samples (see Figure 9). Because of this, we decided that we could not rely on THetA’s copy number calls, so we instead simply removed all SSMs in a location where BIC-seq identified possible structural variation. This eliminated most of the SSMs identified, leaving only 62 SSMs from the original 4,344. Despite this small number of SSMs, our algorithm was still able to identify the correct number of populations and captured the changing composition of the samples. Also, the inferred SSM content of each cluster was identical in the three separate runs.

### Chronic lymphocytic leukemia

**Figure 10:**
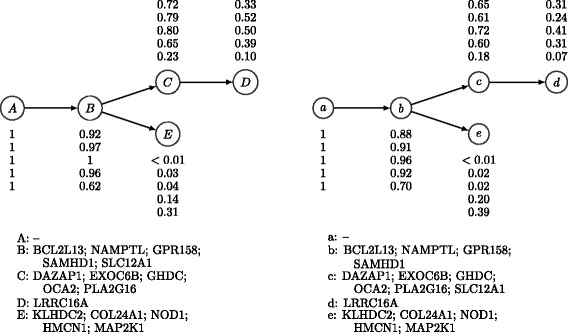
**Expert-generated and inferred phylogenies for patient CLL077 with chronic lymphocytic leukemia.** Left: The expert-generated phylogeny based on targeted deep-sequencing data. Right: The phylogeny inferred by PhyloWGS on allele frequencies of the same SSMs found using WGS. The subclonal lineage population frequencies for the five samples and the SSM assignments of lineages are also shown in the figure. SSM, simple somatic mutation; WGS, whole-genome sequencing.

Next, we applied PhyloWGS to data from patient CLL077 extracted from Supplementary Table 7 from a paper describing a chronic lymphocytic leukemia dataset [11] (available as accession [EGAD:00001000972]). For this patient, five tumor samples were collected over the course of treatment. We note that our method does not assume or use any temporal relationships in multiple sample data and could equally be applied to multiple samples collected simultaneously. We have previously reported experiments using the targeted resequencing data with an average read depth of 100,000 × at 17 identified SSMs [17]; instead we now use the data from WGS for that same set of mutations, with average read depth of 40 ×. By examining the number of reference and variant alleles it was clear that the mutation in gene *SAMHD1* was at a location that was homozygous in the cancerous subpopulation it was part of. This is because the proportion of variant reads was far above 50% (the expected variant allele proportion for a heterozygous SSM present in every cell of the sample). We simulated the data that a CNV algorithm would find by assuming that the copy number at that location was one in a CNV-defined subpopulation and that the proportion of cells in that population was the same as implied by halving the proportion of variant alleles. After running PhyloWGS on these data, we compared the maximum data likelihood tree with the expert-generated tree found using a semi-manual method and targeted resequencing data (Figure 10). The two trees are nearly identical with the exception of assigning a single SSM to a child of the subpopulation where it is found in the expert tree. In Additional file 2, we show the top 50 sampled trees, ranked based on their posterior probabilities.

### Breast tumor

**Figure 11:**
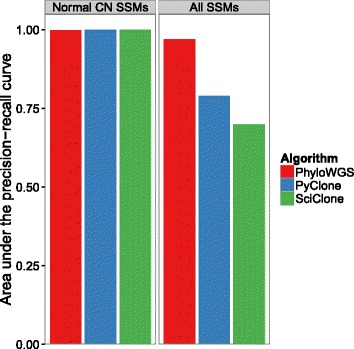
**Subclonal reconstruction algorithms applied to breast tumor PD4120.** Left: Area under the precision–recall curve (AUPRC) for PhyloWGS, PyClone and SciClone when looking at SSMs in areas of normal copy number. Right: AUPRC for PhyloWGS, PyClone and SciClone when looking at SSMs in areas of altered and normal copy number. CN, copy number; SSM, simple somatic mutation.

We analyzed data from WGS at 288 × coverage for tumor PD4120a, first reported in [26] and re-analyzed in [15] (available as accession [EGAD:00001000138]). We confined our analysis to SSMs in genomic regions where THetA and the original analysis agreed on the copy number status of the genome (chr 3,4q,5,10,13,16q,17,19 and 20). These regions contain a total of 26,029 SSMs, of which 4,739 were in regions affected by clonal copy number changes and 2,171 were in regions affected by subclonal copy number changes. We then ran PhyloWGS, PyClone and SciClone on SSMs in regions of normal copy number and on SSMs in regions of both altered and normal copy number. PyClone uses a non-phylogenic correction for copy number alterations and SciClone performs no correction. Based on the semi-manual clustering from [26], we identified those mutations assigned to clusters D, C and B with high probability, which we used as our gold standard for clustering. We then compared the AUPRC for all three algorithms on the two datasets (see Figure 11). All three algorithms have very similar performance when only looking at SSMs in normal regions (Figure 11, left panel). PhyloWGS continues to have very high performance when SSMs in regions of copy number alterations are included, while both PyClone and SciClone have much worse performance than PhyloWGS (Figure 11, right panel).

## Discussion

Our work makes two important contributions to the burgeoning field of subclonal reconstruction. First, we provide the first automated method that integrates SSM and CNV data in the reconstruction of tumor phylogenies. This is an important innovation; previous methods either ignore the impact that CNVs have on SSM allele frequencies [16,21], or assume that the CNVs affect all (and only) the cells that contain the SSM [17-19]. These assumptions can lead to incorrect inferences about the population frequency of SSMs because how a CNV affects the allele frequency of an SSM depends on its phylogenic relationship with the SSM. Many of the insights about how to integrate SSM and CNV data appear in [26]; our work here extends and formalizes these seminal observations while also providing an automated method for phylogenic reconstruction. A further advantage of combining SSMs and CNVs in the phylogenic reconstruction is that CNVs overlapping the SSM locus can provide further constraints on the tree structure than are provided by SSM frequency alone, and we described one case where it is possible unambiguously to infer branching when an amplification of a SSM-containing locus does not lead to a large increase in the SSM allele frequency.

Second, we show that given typical WGS read depths, SSM-based methods are able to reconstruct tumor phylogenies accurately, and detect and assign SSMs for at least six subpopulations. Previously, it was not clear to what extent this reconstruction would be possible and no automated reconstructions with more than two cancerous subpopulations based on WGS data had been described. Furthermore, we demonstrate the importance of phylogenic correction of VAFs of SSMs that occur in loci affected by copy number changes when performing subclonal reconstruction. Specifically, we presented results for a breast cancer benchmark where methods that do not use PhyloWGS’s phylogenic correction perform much worse at recovering subpopulations. Finally, we report examples of accurate subclonal reconstruction for cancer populations with highly reordered chromosomes solely on the basis of SSM frequencies in the regions of normal copy number. For these same data, a state-of-the-art CNV-based method failed to perform the reconstruction.

The current version of PhyloWGS relies on preprocessing the sequencing data with a CNV-based method for subclonal reconstruction. This is because it assumes that the initial population frequency *ϕ*_*i*_ and copy number data *C*_*i*_ are already available for the CNVs; furthermore, for amplifications, *C*_*i*_>2, it requires *C*_*i*_ to be separated into the relative number of each of the two copies, i.e., $\{{C_{i}^{m}}, {C_{i}^{p}}\}$. It does not, however, require the SSMs to be phased; in other words, it does not need to know whether the SSMs occurred on the maternal or paternal copy of the chromosome. New CNV-based methods provide $\{{C_{i}^{m}}, {C_{i}^{p}}\}$ [38]; our work anticipates further developments in subclonal CNV callers. If used with a method that cannot decompose copy number changes into changes in the maternal and paternal loci, PhyloWGS can be restricted to regions of copy number loss (i.e. *C*_*i*_<2), where there is only one possible breakdown. Note that this decomposition is only necessary when an SSM precedes a CNV on the same branch of a phylogeny.

We also note that PhyloWGS does not require the CNV-based preprocessing to be able to detect all of the subclonal populations, and we have shown that PhyloWGS can detect additional populations either defined completely by SSMs or that were not detected by CNV-based methods. This is particularly important because recent CNV-based methods are limited to a maximum of two cancerous populations and those that can detect >1 cancerous subpopulation do so by relying on a strong parsimony assumption. If invalid, this assumption can lead to large errors in subclonal reconstruction because it selects branching phylogenies over chain phylogenies that are equally well supported by the data.

Indeed our results suggest an alternative strategy for combining SSMs and CNVs in subclonal reconstruction. Regions unaffected by CNVs can be relatively easily detected using methods such as BIC-seq [37]. Even in highly rearranged cancer genomes, there are often non-negligible regions of normal copy number and we have shown that we can achieve reasonably accurate subclonal reconstructions using the limited number of SSMs in regions of normal copy number in the TCGA benchmark.

In the final stages of preparing this manuscript, a new method, cloneHD [27], was published. Like PhyloWGS, this method combines both SSMs and CNVs in subclonal reconstruction and does so using WGS data from single and multiple samples. However, unlike PhyloWGS, cloneHD does not explicitly perform phylogenic reconstruction, so it is unable to account fully for the phylogenic relationship among SSMs and CNVs when analyzing SSM allele frequency. As such, it is not clear to us that it can correctly solve the subclonal reconstruction problem posed in Figure 1. The cloneHD manuscript also does not extend the limits of WGS-based subclonal reconstruction as none of the examples reconstruct more than two cancerous subpopulations from a single sample. Finally, cloneHD appears to rely on the strong parsimony assumption to assess subclonal genotypes, and only reports a single reconstruction, obscuring the uncertainty involved. However, cloneHD does appear to be an interesting and powerful method and we hope that future work can compare the merits and drawbacks of these alternate approaches to subclonal reconstruction.

## Conclusions

We have presented a new method, PhyloWGS, which reconstructs tumor phylogenies and characterizes the subclonal populations present in a tumor sample using both SSMs and CNVs. Our method takes as input SSM variant and germ-line read counts, as well as estimates of population frequencies and copy number for each CNV. PhyloWGS groups the SSMs and CNVs into subpopulations, and estimates the population frequencies and the phylogenic relationship of these subpopulations. PhyloWGS is based on a generative probabilistic model of allele frequencies that incorporates a non-parametric Bayesian prior over trees. The output of PhyloWGS consists of samples from this distribution generated through MCMC and we report the tree that maximizes the likelihood of the data found during the sampling run, if a single point estimate is required. However, unlike our previous PhyloSub method [17], PhyloWGS includes CNVs in its subclonal reconstruction and, in doing so, can correctly account for the effect of CNVs on the VAF of overlapping SSMs. PhyloWGS also runs more than 50 times faster than PhyloSub, making it feasible to apply it to the thousands of SSMs that are found through WGS.

We have applied PhyloWGS to real and simulated data from WGS of tumor samples to demonstrate that subclonal populations can be reliably reconstructed based solely on SSMs from medium-depth sequencing (30 to 50 ×). We have also used PhyloWGS to solve correctly a simulated subclonal reconstruction problem that neither an SSM-based nor a CNV-based method could solve alone, and to reconstruct the phylogeny and subclonal composition of a highly rearranged sample for which a CNV-based method fails. We also demonstrated that when applied to WGS of time-series samples from a chronic lymphocytic leukemia patient, PhyloWGS recovers the same tumor phylogeny previously reconstructed by applying PhyloSub and a semi-manual method to data from deep targeted resequencing. Finally, we demonstrated state-of-the-art performance in subclonal reconstruction on a breast tumor sample, highlighting the advantages of performing phylogenetic CNV correction. Our work thus greatly expands the range of tumor samples for which subclonal reconstruction is possible, enabling widespread use of automated subclonal reconstruction for medium-depth WGS sequencing experiments.

## Materials and methods

### PhyloSub model

Our probabilistic model for read count data is based on PhyloSub [17]. For each SSM that is detected by high-throughput sequencing methods, cells containing the SSM are referred to as the variant population and those without the variant as the reference population. Let *a*_*i*_ and *b*_*i*_ denote the number of reads matching the reference allele A and the variant allele B, respectively, at position *i*, and let *d*_*i*_=*a*_*i*_+*b*_*i*_. Let ${\mu _{i}^{r}}$ denote the probability of sampling a reference allele from the reference population. This value depends on the error rate of the sequencer. Let ${\mu _{i}^{v}}$ denote the probability of sampling a reference allele from the variant population, which is set to 0.5 if there are no CNVs; in other words, the SSM is assumed to affect only one of the two chromosomal locations. Let $\tilde {\phi }_{i}$ denote the fraction of cells from the variant population, i.e., the SSM population frequency at position *i*, and $1-\tilde {\phi }_{i}$ denote the fraction of cells from the reference population at position *i*. Let DP(*α*,*H*) denote the Dirichlet process (DP) prior with base distribution *H* and concentration parameter *α*. Samples from the DP are used to generate the SSM population frequencies $\{\tilde {\phi _{i}}\}$. The observation model for allelic counts has the following generative process:

(1) $$ \begin{aligned} & \mathcal{G} \sim \text{DP}(\alpha,H); \quad \tilde{\phi}_{i} \sim \mathcal{G}; \\ & a_{i} \mid d_{i},\tilde{\phi}_{i},{\mu^{r}_{i}},{\mu^{v}_{i}} \sim \text{Binomial}(d_{i},(1-\tilde{\phi}_{i}){\mu^{r}_{i}}+\tilde{\phi}_{i} {\mu_{i}^{v}}). \end{aligned}  $$

The posterior distribution of $\tilde {\phi }_{i}$ is $p(\tilde {\phi }_{i} \mid a_{i},d_{i},{\mu _{i}^{r}},{\mu _{i}^{v}}) \propto p(a_{i} \mid d_{i},\tilde {\phi }_{i},{\mu _{i}^{r}},{\mu _{i}^{v}}) p(\tilde {\phi }_{i})$.

The Dirichlet process prior DP(*α*,*H*) in the observation model of allelic counts (1) is useful for inferring groups of mutations that occur at the same SSM population frequency [12,18]. Furthermore, being a non-parametric prior, it allows us to avoid the problem of selecting the number of groups of mutations *a priori*. However, it cannot be used to model the clonal evolutionary structure, which takes the form of a rooted tree. To model this, we use the tree-structured stick-breaking process prior [46] denoted by TSSB(*α*,*γ*,*H*). The parameters *α* and *γ* influence the height and width of the tree, respectively, and are similar to the concentration parameter in the Dirichlet process prior. Let $\{\phi _{k}\}_{k=1}^{K}$ denote the set of unique frequencies in the set $\{\tilde {\phi }_{i}\}_{i=1}^{N}$, where *K* is the number of subclones or nodes in the tree. In other words, multiple elements in the set $\{\tilde {\phi }_{i}\}_{i=1}^{N}$ will take on the same value from the set $\{\phi _{k}\}_{k=1}^{K}$ of unique frequencies. The prior/base distribution *H* of the SSM population frequencies is the uniform distribution Uniform(0,1) for the root node and $\text {Uniform}(0,\phi _{\text {par}(v)}-\sum _{w \in \mathcal {S}(v)} \phi _{w})$ for any other node *v* in the tree, where par(*v*) denotes the parent node of *v* and $\mathcal {S}(v)$ is the set of siblings of *v*. This ensures that the clonal evolutionary constraints (discussed below) are satisfied when adding a new node to the tree. Given this model and a set of *N* observations $\{(a_{i},d_{i},{\mu _{i}^{r}},{\mu _{i}^{v}})\}_{i=1}^{N}$, the tree structure and the SSM population frequencies $\{\tilde {\phi }_{i}\}$ are inferred using MCMC sampling (see PhyloSub [17] for further details).

Given the current state of the tree structure, we sample SSM population frequencies in such a way that the SSM population frequency *ϕ*_*v*_ of every non-leaf node *v* in the tree is greater than or equal to the sum of the SSM population frequencies of its children. To enforce this constraint, we introduce a set of auxiliary variables {*η*_*v*_}, one for each node, that satisfy $\sum _{v} \eta _{v} =1$, and rewrite the observation model for allelic counts 1 explicitly in terms of these variables resulting in the following posterior distribution:

(2) $$  p(\tilde{\eta}_{i} \mid a_{i},d_{i},{\mu_{i}^{r}},{\mu_{i}^{v}}) \propto p(a_{i} \mid d_{i},G_{i} = g,\tilde{\eta_{i}},{\mu_{i}^{r}},{\mu_{i}^{v}}) p(\tilde{\eta}_{i}),  $$

where we have used $\{\tilde {\eta }_{i}\}$ to denote the auxiliary variables for each SSM. The prior/base distribution of the auxiliary variables is defined such that it is 1 for the root node and Uniform(0,*η*_par(*v*)_) for any other node *v* in the tree. When a new node *w* is added to the tree, we sample *η*_*w*_∼Uniform(0,*η*_par(*w*)_) and update *η*_par(*w*)_←*η*_par(*w*)_−*η*_*w*_. This ensures that $\sum _{v} \eta _{v} =1$. This change is crucial as it allows us to design a Markov chain that converges to the stationary distribution of {*η*_*v*_}. The SSM population frequency for any node *v* can then be computed via $\phi _{v}= \eta _{v} + \sum _{w \in \mathcal {D}(v)} \eta _{w} = \eta _{v} + \sum _{w \in \mathcal {C}(v)} \phi _{w}$, where $\mathcal {D}(v)$ and $\mathcal {C}(v)$ are the sets of all descendants and children of node *v* respectively. This construction ensures that the SSM population frequencies of mutations appearing at the parent node are greater than or equal to the sum of the frequencies of all its children. We use the Metropolis–Hastings algorithm [47] to sample from the posterior distribution of the auxiliary variables $\{\tilde {\eta _{i}}\}$ (2) and derive the SSM population frequencies from these samples by selecting the sampled set of population frequencies with the highest likelihood. We use an asymmetric Dirichlet distribution as the proposal distribution.

### Integrating copy number variation data into PhyloSub

The focus of our new method, PhyloWGS, is integrating SSM frequencies with existing CNV-based subclonal reconstructions. As mentioned above, our algorithm takes as input a set of SSMs along with their allele frequencies, expressed for each SSM *i*, as the number of reads at the locus supporting either the SSM (*b*_*i*_) or the reference allele (*a*_*i*_). We also allow our algorithm to take a set of inferred copy number changes, where for each change *j*, the input provides the new copy number *C*_*j*_ as well as the proportion of the population with the change $\tilde {\phi }_{j}$. In some cases, we also require the breakdown of *C*_*j*_ into the new number of maternal (${C_{j}^{m}}$) and paternal (${C_{j}^{p}}$) copies of the locus (see below for details). If this breakdown is not available, we can restrict our attention to CNVs for which *C*_*j*_<2 because in these cases, there is only one possible breakdown. Also, in the absence of a paternal/maternal breakdown, we should still be able, in theory, to assign SSMs with overlapping CNVs with *C*_*j*_>2 to specific populations once the phylogeny and subclonal populations have been defined using SSMs and CNVs in regions of *C*_*j*_≤2.

Below, we describe the rules, based on the ISA, that we use to determine the relationship between the population frequency of an SSM $\tilde {\phi }_{i}$ and its observed VAF (*b*_*i*_/*d*_*i*_). When the SSM does not overlap a region that has a predicted CNV in any cell in the tumor population, then the predicted allele frequency is simply half of the modeled population frequency. We also describe the method by which we transform each CNV *j* into a pseudo-SSM to be included in the phylogeny.

#### If copy number variations do not overlap with any simple somatic mutation

If a CNV occurs in a region without any SSMs, we generate a pseudo-SSM for the CNV *j*, which is represented in the model as a heterozygous, binary somatic mutation with a read depth that reflects the uncertainty in the provided population frequency $\tilde {\phi }_{j}$ for the CNV. Specifically, we generate reference and variant read counts, *a*_*j*_ and *b*_*j*_, respectively, such that the allelic frequency *b*_*j*_/(*a*_*j*_+*b*_*j*_) is approximately equal to $\tilde {\phi _{j}} / 2$ and the total number of reads *a*_*j*_+*b*_*j*_ is selected based on the evidence supporting the CNV. Generating this pseudo-SSM allows the CNV to be treated like any other SSM in the phylogeny model.

#### If copy number variations overlap with simple somatic mutations

If a structural variant occurs in a region with an SSM *i*, this complicates the relationship between the proportion of cells that contain the SSM and the expected number of reads because cells with the CNV will have more (or fewer) than two copies of the locus where the SSM lies. Assuming equal sampling of these regions, the expected proportion of reads without the mutation (*ζ*_*i*_) is always: ${{N_{i}^{r}}}/{({N_{i}^{r}}+{N_{i}^{v}})}$ where ${N_{i}^{r}}$ is the number of copies of the locus that have the reference allele and ${N_{i}^{v}}$ is the number of copies of the locus with the variant allele. To account for sequencing error we define *ε* as the probability of reading the reference allele when the locus contains the variant allele and vice versa. The expected proportion of reads containing the reference allele is then:

$$\begin{aligned} \zeta_{i} = \frac{{N_{i}^{r}}(1-\epsilon) + {N_{i}^{v}} \epsilon}{{N_{i}^{r}} + {N_{i}^{v}}}. \end{aligned} $$

Looking at a tumor sample with multiple populations and without structural variations, if each population *u* is present with proportion *η*_*u*_ and where ${s_{i}^{u}}$ is 1 if population *u* contains the SSM *i* and 0 otherwise, then ${N_{i}^{r}} = 2 \times \sum _{u} \eta _{u} (1-{s_{i}^{u}}) +\sum _{u} \eta _{u} {s_{i}^{u}}$ and ${N_{i}^{v}} = \sum _{u} \eta _{u} {s_{i}^{u}}$. This is equivalent to an algorithm that looks at each population and performs the following update. If the population *u* contains the SSM *i* then

$$\begin{aligned} {N_{i}^{r}} &\leftarrow {N_{i}^{r}} + \eta_{u}, \\ {N_{i}^{v}} &\leftarrow {N_{i}^{v}} + \eta_{u}. \end{aligned} $$

If the population does not contain the SSM then:

$$\begin{aligned} {N_{i}^{r}} &\leftarrow {N_{i}^{r}} + 2\eta_{u}, \\ {N_{i}^{v}} &\leftarrow {N_{i}^{v}} + 0. \end{aligned} $$

To take into account CNVs requires a more complex procedure. For each population, for each SSM, the number of reference and variant alleles depends on the copy number of the locus *C*_*i*_ and, potentially, the number of maternal (${C_{i}^{m}}$) and paternal (${C_{i}^{p}}$) copies of the locus *as well as* the evolutionary relationship between the SSM and the CNV. The ISA does not apply for CNVs, adding a further level of complexity, because multiple CNVs at the same locus are possible. For each population, the CNV that affects its contribution to the number of reference and variant genomes can be found by ascending the evolutionary tree towards the root. The first CNV found in this ascent is the CNV relevant for the population. If no CNV is found, then the population is not affected by a CNV. For each population there are five possible situations:

1. The population does not contain the SSM and is not affected by a CNV.
2. The population does not contain the SSM but is affected by a CNV.
3. The population contains the SSM but is not affected by a CNV.
4. The population contains the SSM and is affected by a CNV, and the SSM occurred after the CNV.
5. The population contains the SSM and is affected by a CNV, and the CNV occurred after the SSM.

If a population does not contain the SSM, then even if a copy-number change has occurred (Cases 1 and 2), the update rule is:

$$\begin{aligned} {N_{i}^{r}} &\leftarrow {N_{i}^{r}} + \eta_{u} C_{i}, \\ {N_{i}^{v}} &\leftarrow {N_{i}^{v}} +0. \end{aligned} $$

If a population contains the SSM and the SSM occurred after a copy-number change (or there was no copy-number change) (Cases 3 and 4) then there is a single copy of the mutated genome and the remainder are reference, so the the update rule is:

$$\begin{aligned} {N_{i}^{r}} &\leftarrow {N_{i}^{r}} + \eta_{u} \times \max(0,C_{i}-1), \\ {N_{i}^{v}} &\leftarrow {N_{i}^{v}} + \eta_{u}. \end{aligned} $$

If a population contains the SSM and the SSM occurred before the copy-number change (Case 5) then there are two possibilities, the SSM is on the maternal copy or the paternal copy. If the SSM is on the maternal copy, the update rule is:

$$\begin{aligned} {N_{i}^{r}} &\leftarrow {N_{i}^{r}} + \eta_{u} {C_{i}^{p}}, \\ {N_{i}^{v}} &\leftarrow {N_{i}^{v}} + \eta_{u} {C_{i}^{m}}. \end{aligned} $$

If, however, the SSM is on the maternal copy, the update rule is:

$$\begin{aligned} {N_{i}^{r}} &\leftarrow {N_{i}^{r}} + \eta_{u} {C_{i}^{m}}, \\ {N_{i}^{v}} &\leftarrow {N_{i}^{v}} + \eta_{u} {C_{i}^{p}}. \end{aligned} $$

Note that the breakdown of *C*_*i*_ into ${C_{i}^{m}}$ and ${C_{i}^{p}}$ is only required if the CNV occurs after the SSM on the same branch.

Now that we can calculate ${N_{i}^{r}}$ and ${N_{i}^{v}}$, the observation model for the allelic counts has the following generative process (cf. 1):

(3) $$ \begin{aligned} & \mathcal{G} \sim \text{TSSB}(\alpha,\gamma,H); \quad \tilde{\eta}_{i} \sim \mathcal{G}; \\ & a_{i} \mid d_{i},\tilde{\eta}_{i},\epsilon \sim \text{Binomial}\left(d_{i},\frac{{N_{i}^{r}} (1- \epsilon) + {N_{i}^{v}} \epsilon}{{N_{i}^{v}} + {N_{i}^{r}}}\right). \end{aligned}  $$

Note that in some circumstances, a SSM can be placed on a particular copy of the chromosome by looking for reads that cover the SSM and nearby heterozygous germ-line mutations. If this is not possible, then the likelihood of *a*_*i*_ is the average of two likelihoods: the likelihood of the SSM occuring on the maternal genome and the likelihood of the SSM occuring on the paternal genome.

### Extension to multiple samples

Our model can be easily extended to multiple tumor samples. We make no assumptions regarding the time when the samples were collected, so this extension is equally applicable to multiple samples collected simultaneously (e.g. as in [2]) or over a period of time as in [11]. We allow the tree-structured stick-breaking process prior to be shared across all the samples. The main technical difference between the single and the multiple sample models lies in the sampling procedure for SSM population frequencies. In the multiple sample model, we ensure that the clonal evolutionary constraints are satisfied separately for each tumor sample and then make a global Metropolis–Hastings move based on the product of posterior distributions across all the samples (cf. 2).

### Markov chain Monte Carlo settings

In all the MCMC experiments, we fix the number of MCMC iterations to 2,500 and use a burn-in of 100 samples. We also fix the number of iterations in the Metropolis–Hastings algorithm to 5,000 and set the scaling factor for the Dirichlet proposal distribution to 100 (see PhyloSub paper [17]). We use the CODA R package [48] for MCMC diagnostics to monitor the convergence of the samplers using the complete-data log likelihood traces and the corresponding autocorrelation function.

### Sequencing error

It is becoming increasingly clear that sequencing error is not uniform across the genome and different trinucleotide sequences result in different sequencing error rates [49]. While the precise nature of these differences is not yet fully known, PhyloWGS allows the user to input a different sequencing error rate for each mutation.

## Additional files

Additional file 1
**Supplementary figures.** The figure in this file shows mean co-clustering matrices for simulations with four populations (three cancerous), where the AUPRC is 0.98 **(A)**, 0.90 **(B)**, 0.80 **(C)** and 0.65 **(D)**. Rows and columns correspond to individual SSMs. For visibility, the matrix has been randomly subsampled to 150 SSMs from the 600 SSMs used in the simulation. Pixel color indicates co-clustering probability.

Additional file 2
**Supplementary figure.** This file contains the top 50 sampled trees along with their posterior probabilities for the CLL077 data.

## Abbreviations

AUPRC: area under the precision–recall curve
CNV: copy number variation
DP: Dirichlet process
ISA: infinite sites assumption
MCMC: Markov chain Monte Carlo
SSM: simple somatic mutation
VAF: variant allelic frequency
WGS: whole-genome sequencing
