# Supplementary material for: PhyloWGS: Reconstructing subclonal composition and evolution from whole-genome sequencing of tumors
Source: Genome Biol. 2015 Feb 13;16(1):35. doi: 10.1186/s13059-015-0602-8 (PMC4359439; doi:10.1186/s13059-015-0602-8)
Supplement: Additional file 1 — Supplementary figures. The figure in this file shows mean co-clustering matrices for simulations with four populations (three cancerous), where the AUPRC is 0.98 (A), 0.90 (B), 0.80 (C) and 0.65 (D). Rows and columns correspond to individual SSMs. For visibility, the matrix has been randomly subsampled to 150 SSMs from the 600 SSMs used in the simulation. Pixel color indicates co-clustering probability. [file 13059_2015_602_MOESM1_ESM.zip › 16.pdf]

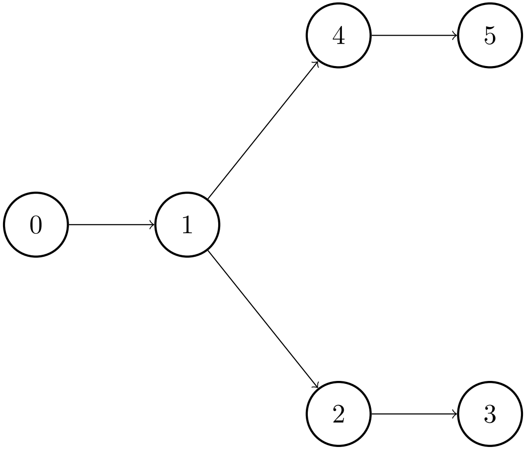

Node	Mutations	Clonal frequencies				
0	–	1.0 ± 0.0	1.0 ± 0.0	1.0 ± 0.0	1.0 ± 0.0	1.0 ± 0.0
1	GPR158, NAMPTL, , BCL2L13	0.9 ± 0.028	0.896 ± 0.032	0.971 ± 0.014	0.942 ± 0.018	0.725 ± 0.049
2	EXOC6B, DAZAP1, GHDC, PLA2G16, SLC12A1, OCA2, SAMHD1	0.662 ± 0.061	0.606 ± 0.06	0.669 ± 0.061	0.583 ± 0.054	0.216 ± 0.041
3	LRRC16A	0.32 ± 0.094	0.358 ± 0.107	0.259 ± 0.078	0.277 ± 0.078	0.061 ± 0.035
4	MAP2K1	0.097 ± 0.033	0.136 ± 0.061	0.123 ± 0.054	0.239 ± 0.046	0.378 ± 0.052
5	HMCN1, NOD1, COL24A1, KLHDC2	0.02 ± 0.009	0.042 ± 0.023	0.043 ± 0.022	0.157 ± 0.029	0.269 ± 0.043

Posterior probability: 0.0096
