# Supplementary material for: PhyloWGS: Reconstructing subclonal composition and evolution from whole-genome sequencing of tumors
Source: Genome Biol. 2015 Feb 13;16(1):35. doi: 10.1186/s13059-015-0602-8 (PMC4359439; doi:10.1186/s13059-015-0602-8)
Supplement: Additional file 2 — Supplementary figure. This file contains the top 50 sampled trees along with their posterior probabilities for the CLL077 data. [file 13059_2015_602_MOESM2_ESM.pdf]

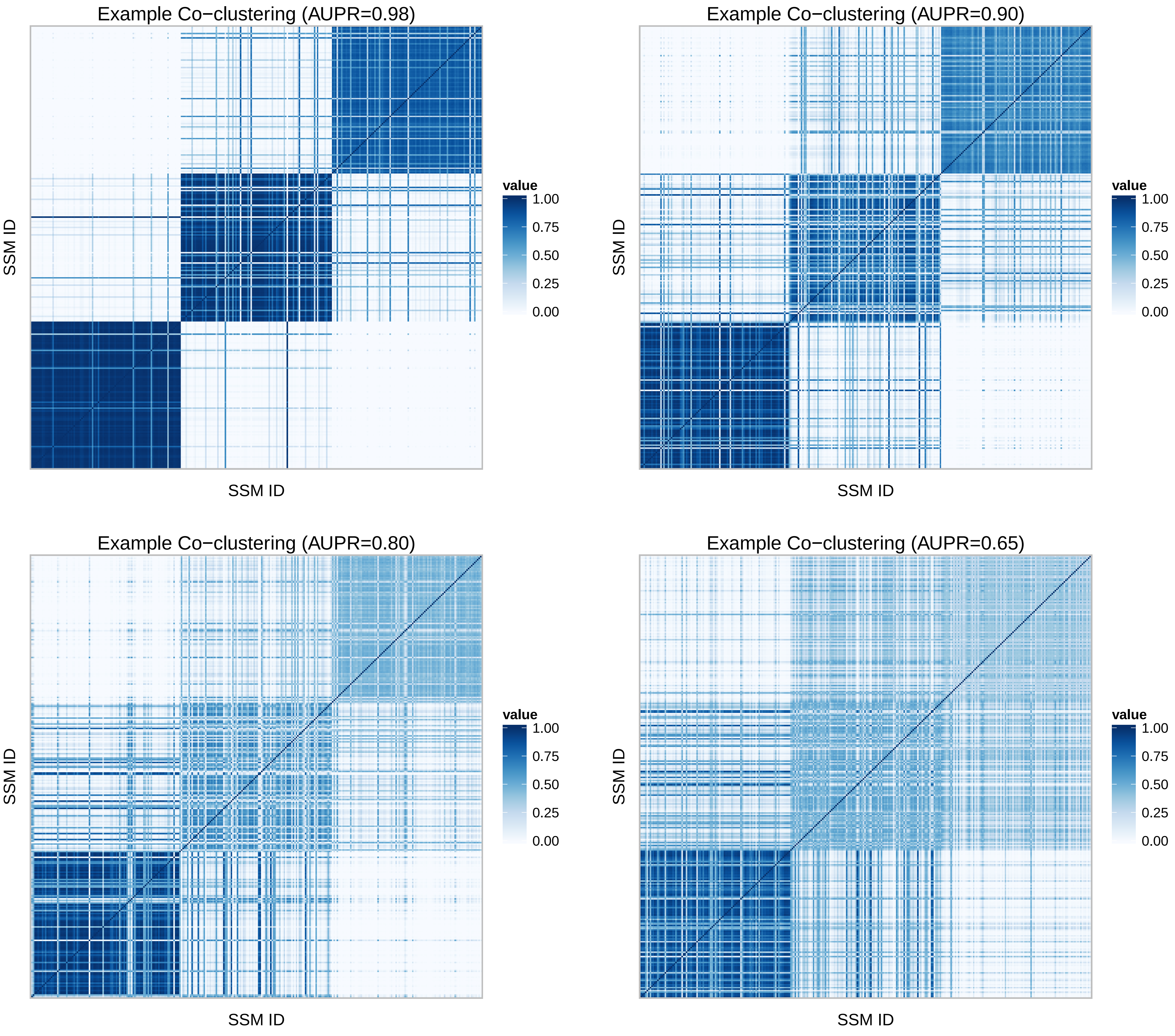

Example Co−clustering (AUPR=0.98)
Example Co−clustering (AUPR=0.90)
Example Co−clustering (AUPR=0.80)
Example Co−clustering (AUPR=0.65)
SSM ID
SSM ID
value
1.00
0.75
0.50
0.25
0.00
